# Crack Growth Simulation of Functionally Graded Materials Based on Improved Bond-Based Peridynamic Model

**DOI:** 10.3390/ma14113032

**Published:** 2021-06-02

**Authors:** Liyi Min, Qiwen Liu, Lisheng Liu

**Affiliations:** 1 Hubei Key Laboratory of Theory and Application of Advanced Materials Mechanics, Wuhan University of Technology, Wuhan 430070, China; liyi_min@whut.edu.cn (L.M.); Liulish@whut.edu.cn (L.L.); 2 Department of Engineering Structure and Mechanics, Wuhan University of Technology, Wuhan 430070, China; 3 State Key Laboratory of Advanced Technology for Materials Synthesis and Processing, Wuhan University of Technology, Wuhan 430070, China

**Keywords:** functionally graded materials, peridynamic method, integral equivalence, crack growth simulation

## Abstract

Functionally graded materials (FGMs) are widely used in the aerospace industry, especially for the thermal protection shields of aerospace vehicles. Studies show that the initiation and expansion of micro-cracks are important factors that adversely affect the service life of these shields. Based on the peridynamic theory of bonds, an improved peridynamic model is proposed in the present study for FGMs. In the proposed model, integral equivalence is applied to calculate the required material parameters. Obtained results reveal that this method can better reflect the gradient change of material properties.

## 1. Introduction

With the development of science and technology in the past few decades, the aerospace industry has developed rapidly. Studies show that during the entire mission of a spacecraft, the materials used may face a very harsh working environment. Accordingly, requirements for the constituent materials of the spacecraft have become increasingly stringent. In this regard, scholars proposed the concept of functionally graded materials (FGMs) made of metals and ceramics. Further investigations reveal that since the composition of these materials changes continuously with spatial position, the material properties on the macroscopic level also change accordingly, thereby eliminating the interface layer between different materials. This characteristic allows FGMs to simultaneously exert the excellent mechanical properties of metals and the high-temperature resistance of ceramics. In practical applications, FGM failures mainly originate from defects in the material and crack propagation. Therefore, it is of extreme importance to investigate the crack propagation phenomenon in FGMs. However, since an FGM is a heterogeneous substance, the material composition may vary spatially. Accordingly, it is an enormous challenge to analyze crack propagation in FGMs.

In the classical continuum theory, spatial partial differential equations are used to describe the material deformation. However, crack propagation is a typical discontinuity problem such that spatial partial derivatives cannot be applied to this problem. Considering the computational difficulties of mathematical methods, it is generally assumed that the FGM is an isotropic substance. Literature review indicates that only a few investigations have been conducted so far on crack propagation in anisotropic FGMs. In this regard, Erdogan et al. systematically studied the static fracture problem of heterogeneous materials [1,2]. Moreover, Jin et al. analyzed the crack propagation problem in homogeneous and FGMs and found that as long as the material parameters were continuous and differentiable, singular fields of the crack tip in these materials were the same [3]. Marur et al. carried out experiments and performed numerical simulations to study the interface fracture of bimaterials and FGMs under different impact loads [4]. Kawasaki et al. applied the controllable burner heating method to simulate the real environment and studied the thermal fracture behavior of FGMs. The obtained results showed that orthogonal cracks form on the top surface of the ceramic layer during the cooling process. Moreover, the gradient layer forms transverse cracks and these cracks propagate, merge, and eventually peel the coating off [5]. Considering the difficulties and high costs of the crack growth experiment in FGMs, it is important to investigate this problem through numerical methods. Meanwhile, conventional experimental methods can hardly reveal the whole cracking process in FGMs, including crack initiation, crack propagation and complete fracture. Currently, different numerical methods, including the finite element method, extended finite element method, boundary element method and meshless method, can be applied to study the FGM crack growth problem. Nishioka et al. presented the concept of mixed-phase simulation with fracture-path prediction mode, in which the crack propagation path is simulated by using the proposed automatic moving finite element method, and the simulation results were in good agreement with the experimental results [6]. Fabbrocino et al. proposed a new methodology to predict dynamic crack propagation under generalized loading conditions, and a numerical model of permission was established based on this method. This model can be used to predict the geometric changes caused by the discontinuous evolution of existing materials; the accuracy of the model was verified by comparison to experimental results. Sensitivity analyses in terms of mesh dependence and time required for the solving procedure were also discussed [7]. Dirik et al. developed a mesh-independent computational algorithm and incorporated this into a commercial finite element software (Abaqus). For verification purposes, experimental crack path trajectories and fatigue life data available in the open literature were compared with computational results [8].

In the classical finite element method, the singularity problem of the spatial derivative is considered as a typical discontinuity problem of crack propagation. Moreover, in the extended finite element method, additional functions are introduced to deal with the crack problem. However, this method has limitations for complex cracks such as multiple cracks and crack bifurcation. Further investigations reveal that the boundary element method has its own shortcomings. More specifically, its scope of action requires a basic solution of the corresponding differential operator as a major premise, which is a challenging prerequisite for heterogeneous materials. Furthermore, the meshless method requires a kernel function, and it also has the limitation of the spatial derivative problem. Recently, Silling proposed the peridynamic (PD) method [9] as a vigorous scheme to solve discontinuous problems, which has attracted many scholars worldwide.

Unlike the finite element method, the PD method does not use partial differential equations, but integral equations. Meanwhile, the material damage is included in the constitutive of the PD method. Accordingly, this method can be applied to simulate the crack propagation problem in the material without the need to set a criterion. Bobaru et al. performed a peridynamics analysis on the dynamic crack growth of brittle materials under different convergence conditions such as m-convergence and δ-convergence and verified the calculated results through experimentation. Accordingly, it was found that the peridynamics method is an effective scheme for simulating the dynamic crack growth problem [10]. Ha et al. used the bond-based PD theory to study the influence of the pre-crack angle on the crack growth of rock-like materials containing pre-crack subjected to uniaxial compressions. The obtained results proved the effectiveness of the PD method [11]. Cheng et al. proposed a near-field dynamic model for FGMs and studied the effects of different parameters, including the material gradient, elastic wave, impact load size and contact time on the fracture behavior of FGMs [12]. Tan et al. proposed a complete thermo-mechanical coupling peridynamics model with a surface correction near cracks, and then applied this model to perform thermodynamic simulations of the FGM plate with thermal insulation cracks [13].

Based on the performed literature survey, we intended to apply the PD theory and propose an improved bond-based peridynamics model. The proposed model will be used to simulate the crack growth of FGMs with the material discretized into scattered points. Therefore, the material properties of the bonds between different particles should be processed and calculated. It should be indicated that the conventional method to do so is to use the average value for processing. However, the material properties of FGMs are not uniform so the average solution method cannot reflect the internal properties of FGMs accurately. The proposed method is expected to reflect the variations of the properties in functionally graded materials.

## 2. Materials and Methods

### 2.1. Bond-Based Peridynamic Basic Theory

**Figure 1:**
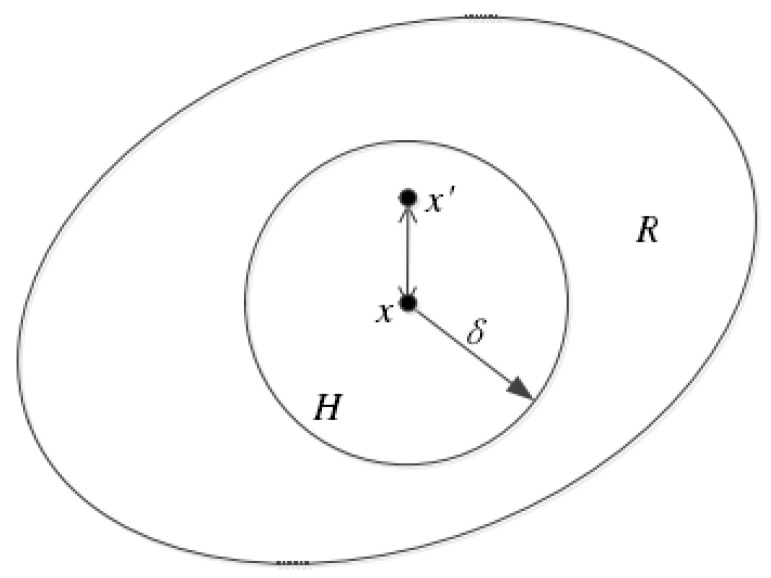
The peridynamic horizon of a material point (*H*), and the whole computing domain (*R*).

Figure 1 shows that in the bond-based PD theory, the object is discretized into several material points, where each material point x only interacts with other material points in the range of δ. This interaction is represented by the bond $\xi =x^{\prime }-x$. It is worth noting that the interaction between two material points is equal in magnitude and opposite in direction. At a certain time, *t*, this motion can be mathematically expressed in the form below:

(1) $$\rho \left(x\right)\ddot{u}\left(x,t\right)=\int_{H_{x}}f\left(x^{\prime },x,t\right)dV_{x^{\prime }}+b\left(x,t\right)$$

where f and u denote the force function between material points and the displacement of the material point at a certain moment, respectively. Moreover, $H_{x}$, ρ and b are the neighborhood range, material density and force on the material point *x*, respectively. $V_{x^{\prime }}$ is the volume of other points in the neighborhood of point *x*.

Each material point only interacts with other material points in its neighborhood. When a point exceeds the neighborhood range δ, then $f\left(x^{\prime },x,t\right)=0$. Silling proposed the following expression for the force function in elastic isotropic materials [14]:

(2) $$f\left(x^{\prime },x,t\right)=\frac{\xi +\eta }{\left|\xi +\eta \right|}cs$$

where $\eta =u^{\prime }-u$ is the relative displacement vector, $\xi =x^{\prime }-x$ is the relative position vector and c represents the micro-elastic modulus of the bond. Moreover, s denotes the elongation of the bond, which is similar to the strain in conventional mechanics. It should be indicated that the parameter c can be obtained through the strain energy density of PD form equal to the strain energy density of the classical elastic theory. The parameter c of an isotropic material subjected to the plane stress is $c=9E/\left(\pi h\delta^{3}\right)$, where E is the Young’s modulus, δ is the size of the neighborhood and h denotes the distance between two material points.

### 2.2. Definition of Bond Break and Damage

In the bond-based peridynamic basic theory, the material failure is judged by the bond break. Currently, two judgment methods have been proposed for the bond break between material points. The first method is to judge based on the critical elongation $s_{0}$, and the second way is to judge based on the critical energy density. In the present study, the critical elongation is selected to judge the failure. To this end, the relative elongation is obtained when the bond between the material points is subjected to force. When the relative elongation exceeds the critical value $s_{0}$, the bond between the material points breaks and the specimen fails. Under this circumstance, there is no longer any interaction between points. The stress of the critical elongation in the plane can be calculated through the following expression:

(3) $$s_{0}=\sqrt{\frac{4\pi G_{0}}{9E\delta }}$$

where $G_{0}$ is the energy release rate during the crack propagation and E is the Young’s modulus. The correlation between the fracture toughness $K_{IC}$, the critical energy release rate $G_{0}$ and the Young’s modulus of the material is as follows:

(4) $$G_{0}=\frac{{K_{IC}}^{2}}{E}$$

The local damage of the material point can be judged by the following damage coefficient:

(5) $$\mu \left(x^{\prime },x,t\right)=\{\begin{array}{c} 1\;\;\;\mathrm{if}\;s\leq s_{0}\\ 0\;\;\;\mathrm{if}\;s>s_{0} \end{array}$$

(6) $$\varphi \left(x,t\right)=1-\frac{\int_{H_{x}}\mu \left(x^{\prime },x,t\right)dV_{x^{\prime }}}{\int_{H_{x}}dV_{x^{\prime }}}$$

when φ=1, the bond between the reference material point and other material points in the domain has been broken. On the contrary, φ=0 indicates that no bond has been broken. Accordingly, the range of the damage coefficient is [0,1].

### 2.3. Improved Bond-Based Peridynamics Model for FGM Analysis

An FGM is a heterogeneous material with variable spatial material composition. Consequently, material properties change along the spatial coordinates. Accordingly, the material properties of an FGM should be considered as spatial functions such as ρ(x,y), E(x,y) and $K_{IC}\left(x,y\right)$. Therefore, the material properties can be expressed in the form below:

(7) $$\beta \left(x,y\right)=\beta \left(\beta_{0},x,y\right)$$

where β can be ρ, E or $K_{IC}$. Similarly, $\beta_{1}$ can be replaced by $\rho_{1}$, $E_{1}$ or $K_{{}_{IC1}}$. (The initial value of the function) In the PD method, the bond material properties between material points $x_{i}\left(x_{i},y_{i}\right)$ and $x_{j}\left(x_{j},y_{j}\right)$ should be obtained in these calculations. Generally, the average of material parameters for two material points can be used to calculate the corresponding PD bond material properties of the FGM. In this regard, the material properties of the bond model solved by the average value can be expressed as:

(8) $$\beta \left(x_{i},x_{j}\right)=0.5\left(\beta \left(x_{i},y_{i}\right)+\beta \left(x_{j},y_{j}\right)\right)$$

The calculated average material parameters are then used to calculate the corresponding micro-modulus and critical elongation in the PD model:

(9) $$c\left(x_{i},x_{j}\right)=\frac{9E\left(x_{i},x_{j}\right)}{\pi h\delta^{3}}$$

(10) $$s_{0}\left(x_{i},x_{j}\right)=\sqrt{\frac{4\pi G_{0}\left(x,\hat{x}\right)}{9E\delta }}$$

However, the material parameters of FGMs do not change uniformly in space. Therefore, although the average value method is convenient to solve, it cannot accurately reflect the variations in the material parameters.

**Figure 2:**
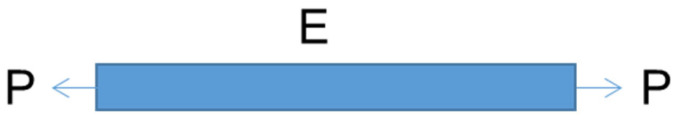
Simplified diagram of bonds between material points.

Figure 2 illustrates a simplified bond model, and shows that its material parameters, such as the Young’s modulus, follow a function along the length direction E(x).

When the two ends of the rod are under tension, the rod elongation can be calculated through the following expression:

(11) $$\Delta l=\int_{0}^{l_{0}}\frac{P}{E(x)A}dx$$

where $l_{0}$ is the distance between two material points and Δl is the amount of change in the distance between the two materials, which can be calculated as follows:

(12) $$\Delta l=\frac{Pl_{0}}{E_{0}A}$$

where $E_{0}$ is the equivalent Young’s modulus of the rod. The equivalent Young’s modulus can then be obtained by combining Equations (11) and (12):

(13) $$E_{0}=\frac{l_{0}}{\int_{0}^{l_{0}}1/E\left(x\right)dx}$$

E(x) is a function reflecting the variations in material parameters. It is an enormous challenge to solve this integral expression theoretically. Therefore, a numerical integration method can be used to solve it. Subsequently, the equivalent parameters between the two material points can be obtained and the corresponding micro-modulus and critical elongation can be solved.

## 3. Model Checking

This section intends to verify the accuracy of the improved PD model established in Section 2. To this end, the crack growth in an organic glass plate and an FGM beam under different loads are simulated.

### 3.1. Simulating the Crack Propagation in a Plexiglass Sheet

#### 3.1.1. Program Verification

**Figure 3:**
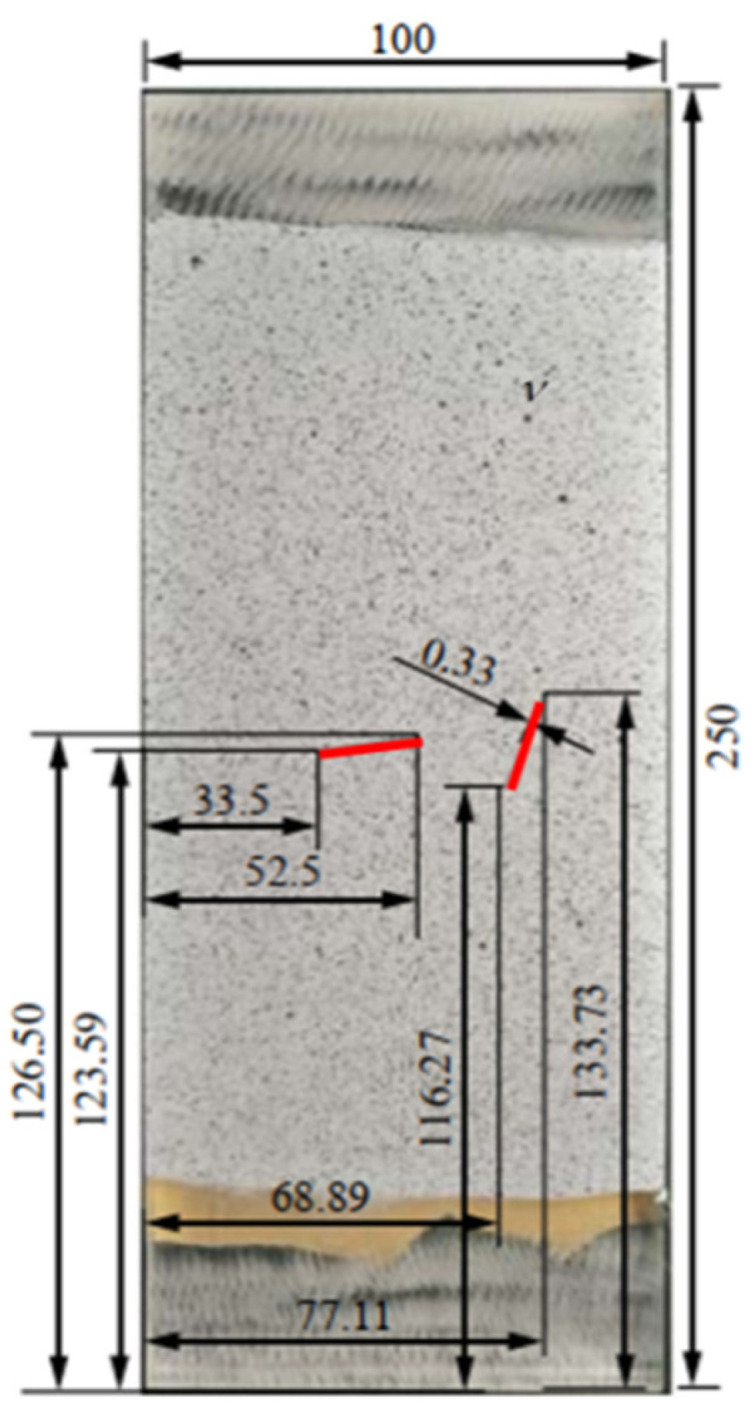
Schematic diagram of the pre-crack position [15].

In order to verify the accuracy of the near-field dynamics program, the crack propagation problem in a rectangular plexiglass sheet with prefabricated cracks under a uniformly distributed load at both ends is simulated. The board is 250 mm long and 100 mm wide, and the position of the pre-crack is shown in Figure 3.

The red solid line in Figure 3 shows the position of the prefabricated crack. Parameters of the plexiglass are presented in Table 1.

**Figure 4:**
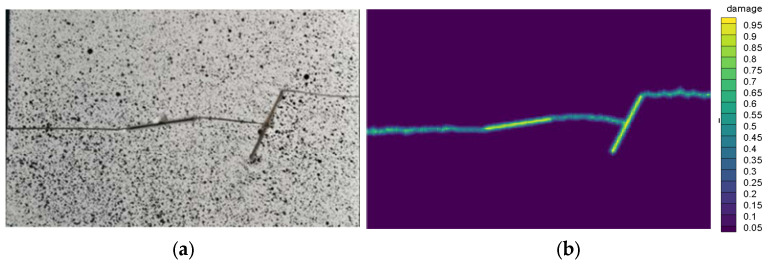
Crack path at complete failure obtained from the (**a**) experiment [16] and (**b**) numerical simulation.

The upper and lower ends of the plate are subjected to a uniform load of 13 MPa, and the particle spacing is 0.0005 m. Moreover, the neighborhood size is set to three times the particle spacing, and the calculation time step is set to 0.1 microseconds. The influence function is defined as follows [16]:

(14) $$g\left(\lambda ,\Delta \right)={\left(1-{\left(\lambda /3\Delta \right)}^{2}\right)}^{2}$$

where λ is the distance between two particles at each moment, and Δ is the particle spacing set in the initial model. Equation (14) is then multiplied by the corresponding force function to get the corresponding particle interaction. Figure 4a,b shows the experimental and calculated final forms of the cracks, respectively.

Comparison of the results from the experiment and numerical simulation reveals that there is a good consistency, indicating that the established method is accurate.

Next, the parameters affecting the accuracy of the PD calculation, including the influence function, neighborhood range and particle distance, are investigated. The main purposes of this investigation are to apply (1) the influence function, (2) different neighborhood ranges and (3) different particle spacing on the crack path of the plexiglass plate. The calculation results are then compared with the experimental results to evaluate the influence of the different parameters on the simulation accuracy and calculation efficiency.

#### 3.1.2. The Effect of the Influence Function on the Calculation Accuracy

**Figure 5:**
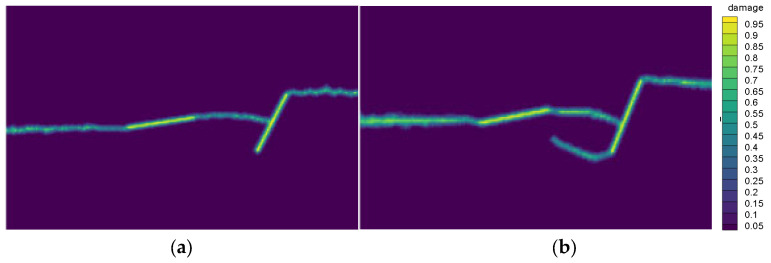
Crack path diagram (**a**) with the influence function, and (**b**) without the influence function.

Within the neighborhood of a particle, the distance between the reference particle and other particles is different, resulting in different interactions between particles. Generally, the longer the distance, the smaller the interaction. In the present study, only the influence function shown in Equation (14) is applied to calculate the crack propagation path of the plexiglass plate with and without the influence function. The obtained results in this regard are shown in Figure 5. Except for the influence function, other material properties and dimensions of the calculation model are the same as those in Section 3.1.1.

Figure 5a,b reveals that the calculated results after setting the influence function are in better agreement with the experiment shown in Figure 4. Accordingly, it is concluded that setting an appropriate influence function can improve the calculation accuracy.

#### 3.1.3. The Influence of the Neighborhood Range on Calculation Accuracy

**Figure 6:**
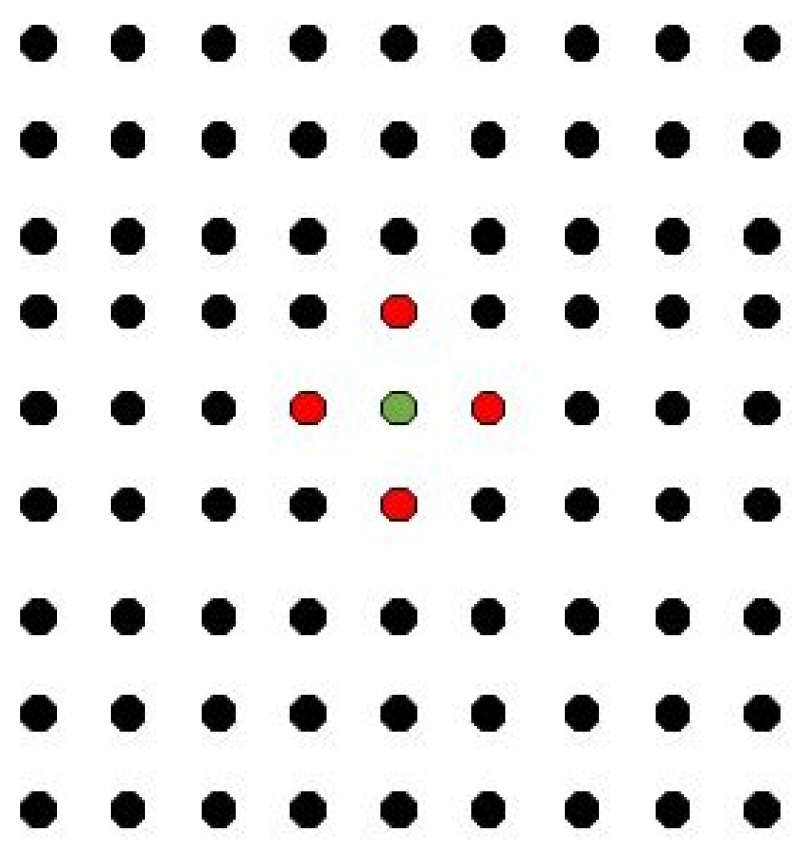
The peridynamic horizon of the single-particle spacing.

In the numerical calculation of the bond-based PD theory, it is necessary to set the size of the neighborhood of the material points to determine the distance at which a material point interacts with other points. For example, when the neighborhood range is set as particle spacing δ=Δd, this means that each particle only interacts with the nearest particle nearby. This issue is shown in the following two-dimensional case (Figure 6).

In this section, the crack paths are calculated in different neighborhoods, including δ=1Δd, $\delta =\sqrt{2}\Delta d$, δ=2Δd, $\delta =\sqrt{5}\Delta d$, $\delta =2\sqrt{2}\Delta d$, δ=3Δd, $\delta =\sqrt{10}\Delta d$, $\delta =\sqrt{13}\Delta d$. It should be indicated that in all calculations, the same model as that in Section 3.1.1 is applied.

**Figure 7:**
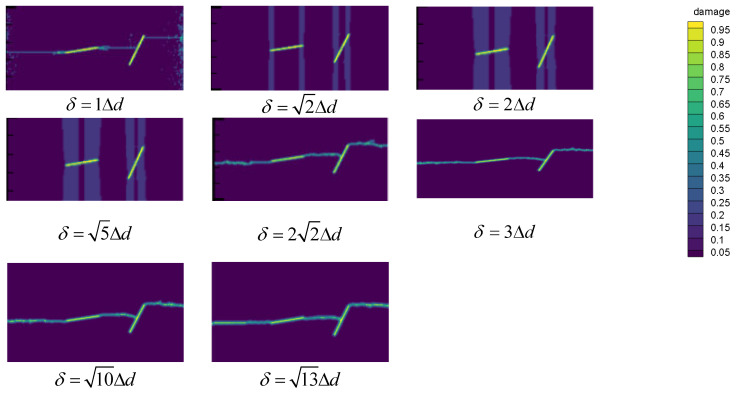
Crack path with different neighborhoods.

Figure 7 shows that the best calculation results can be obtained when the neighborhood range is three times the particle spacing or more. However, as the neighborhood range increases, the corresponding calculation time increases significantly. Accordingly, the neighborhood range three times the particle spacing is considered as the best spacing with the best accuracy and efficiency.

#### 3.1.4. The Effect of the Particle Spacing on the Calculation Accuracy

Studies show that particle spacing greatly affects the numerical calculation results of the PD theory. Generally, the smaller the grid size, the higher the accuracy of the calculation result. However, the grid size cannot be reduced indefinitely because this will greatly increase the calculation time. Therefore, it is necessary to set an appropriate particle spacing in the calculation in order to meet the accuracy and efficiency requirements of the calculation. In this regard, the crack propagation path of the plexiglass plate with different particle spacing, including $\Delta d=2\times 10^{-3}\;m$, $\Delta d=1\times 10^{-3}\;m$ and $\Delta d=5\times 10^{-4}\;m$, are simulated in this section. Note that except for the particle spacing, all parameters are the same as those in Section 3.1.1.

**Figure 8:**
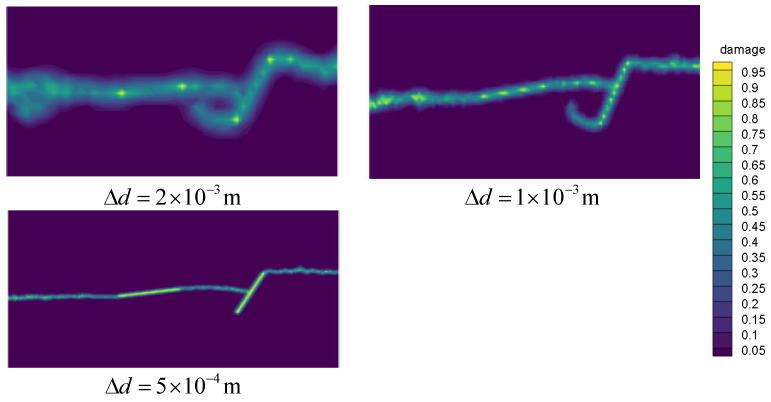
Crack path diagrams with different particle spacing.

Figure 8 indicates that for the particle spacing of $\Delta d=2\times 10^{-3}\;m$ and $\Delta d=1\times 10^{-3}\;m$, the crack path differs from the experiment. On the other hand, when the particle spacing is set to $5\times 10^{-4}\;m$, a good agreement can be achieved with the experiment. Further reduction in the grid size greatly increases the calculation time, thereby adversely affecting the calculation efficiency. Therefore, it is concluded that when the grid size is less than or equal to the order of $10^{-4}\;m$, the accuracy requirements of the calculation can be met.

### 3.2. Simulation of Crack Propagation in FGM Beam under a Four-Point Bending Load

**Figure 9:**
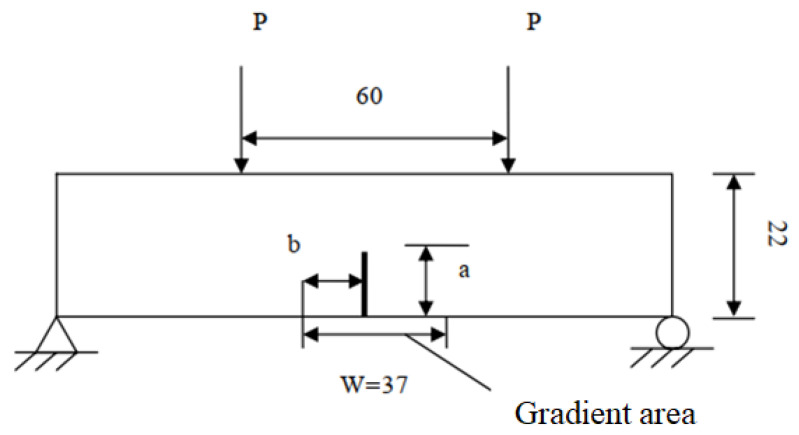
Schematic diagram of the model.

In order to further verify the validity and reliability of the improved PD model, a four-point bending pre-cracked FGM specimen is simulated. The section size of the test specimen is 120 mm × 22 mm. Under the action of a four-point bending load, there is a material transition zone with a length of W = 37 mm in the middle of the specimen. Figure 9 schematically shows the model. The left side of the transition area is pure epoxy, while the right side is pure glass. The initial length of the crack is a = 5.5 mm, b = 14 mm. The particle distance and the calculation time step are set to 0.0025 m and 0.02 ms, respectively. Moreover, the neighborhood radius is three times the particle distance and the load P is 100 kN.

The material parameters of the studied FGM are presented in Table 2.

The material parameters of the gradient region change in the form of a power function as below:

(15) $$\beta \left(x,y\right)=\beta_{0}+\left(\beta_{w}-\beta_{0}\right){\left(\frac{x}{W}\right)}^{4}$$

**Figure 10:**
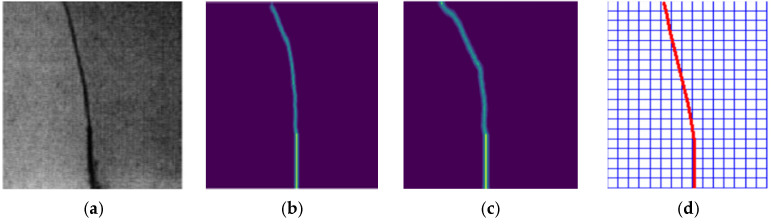
Crack path diagram: (**a**) Experimental result [17], (**b**) calculation results obtained from the improved PD model, (**c**) calculation results from the standard PD model and (**d**) extended finite element calculation results.

Comparison of Figure 10a–d indicates that the calculation results obtained from the proposed model are in good agreement with the experiment.

In order to compare the above crack path more clearly and intuitively, the corresponding data are extracted and a chart is prepared accordingly.

**Figure 11:**
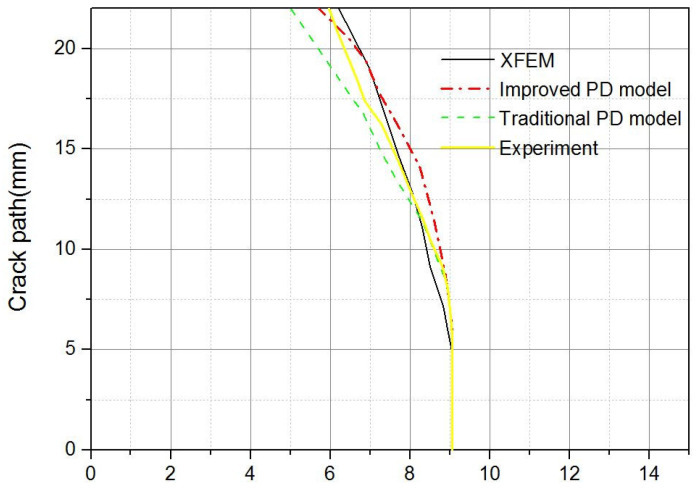
Crack path diagram.

Figure 11 shows that in the early stages of crack propagation, the calculation model in this paper does not reflect the corresponding superiority. However, as the crack continues to grow, it can be observed that the calculation results of the traditional PD model gradually deviate from the experimental results. When the complete specimen is fractured, with the experimental results as the reference point (breaking point at top), the calculation error of the traditional PD model is 16.1% whereas the error of the model in this paper is 4.3%. Therefore, it can be considered that the model proposed in this paper improves the calculation accuracy to a certain extent.

## 4. Conclusions

In the present study, the integral equivalent solution method is applied to propose an improved bond-based PD model and calculate the material parameters of the bond. Compared with the conventional average solution method, the proposed method is more in line with actual material property gradient changes. The proposed model is applied to simulate the dynamic crack growth of ae plexiglass plate and an FGM beam under different loads. The calculation results are then compared with the experiment, and the model accuracy is evaluated. The influence of different PD parameters on the simulation accuracy was discussed, and it is concluded that the best results can be achieved when the affecting parameters are set as follows: 1. the neighborhood range is set to three times the particle spacing, 2. an appropriate influence function should be considered in the calculations and 3. the distance between particles is on the order of 10^−4^ m or less.

It is found that compared with conventional methods, the results of the proposed model are more in line with the experiment.

## Figures and Tables

**Table 1:** Material parameters of plexiglass.

Material Parameter	Plexiglass
Elastic modulus E (GPa)	2.633
Density *ρ* (kg/m^3^)	1200
Fracture toughness *K_IC_* (MPa·m^1/2^)	2.2

**Table 2:** Material parameter table of the FGM.

Material Parameter	Epoxy Resin	Glass
Elastic modulus *E* (GPa)	3	8.6
Density *ρ* (kg/m^3^)	1200	1850
Fracture toughness *K_IC_* (MPa·m^1/2^)	1.2	2.6
